# Mechanistic insights into azo compound back-isomerization from spin-flip time-dependent DFT combined with Marcus theory

**DOI:** 10.1039/d6sc01578f

**Published:** 2026-04-27

**Authors:** Ari Serez, Flavia Aleotti, Pascal Gerbaux, Luca Muccioli, Jérôme Cornil

**Affiliations:** a Laboratory for Chemistry of Novel Materials, University of Mons Place du Parc 23 7000 Mons Belgium Jerome.Cornil@umons.ac.be; b Organic Synthesis and Mass Spectrometry Laboratory, University of Mons Place du Parc 23 7000 Mons Belgium; c Dipartimento di Chimica Industriale “Toso Montanari”, University of Bologna Via Gobetti 85 40129 Bologna Italy

## Abstract

Interest in photosensitive molecules has increased significantly over the past decade, with particular attention given to photoswitchable systems. Among these, azobenzene stands out as a reference compound due to its broad range of applications, in particular for solar energy storage. While the *trans*-to-*cis* photoisomerization has been relatively well characterized, the reverse *cis*-to-*trans* isomerization remains a complex process potentially involving multi-state physics. In this study, we compile recent theoretical advances aimed at modeling this process and introduce, through the spin-flip time-dependent density functional theory (SF-TDDFT) approach combined with the semi-classical Marcus equation, a fast and efficient method to investigate the mechanisms of thermal back-isomerization of azo derivatives. By comparing various exchange–correlation functionals with CASPT2 reference data, we demonstrate that the PBE0(D3BJ) functional provides an accurate description for the non-adiabatic rotational pathway. We successfully reproduce the experimental values (88.6 *vs.* 88.3 kJ mol^−1^ for the experimental enthalpy of activation, and −53.0 *vs.* −50.2 J mol^−1^ K^−1^ for the experimental entropy of activation) for azobenzene, thus motivating the extension of this methodology to other azo derivatives. This approach can be further generalized to a broader class of azo-based photo-switches in future studies.

## Abstract 2

Non-adiabatic rotation modeled by combining spin-flip TDDFT with Marcus theory. This approach characterizes electronic coupling and energy barriers at the singlet–triplet crossing to investigate spin-forbidden pathways.

## Graphical abstract

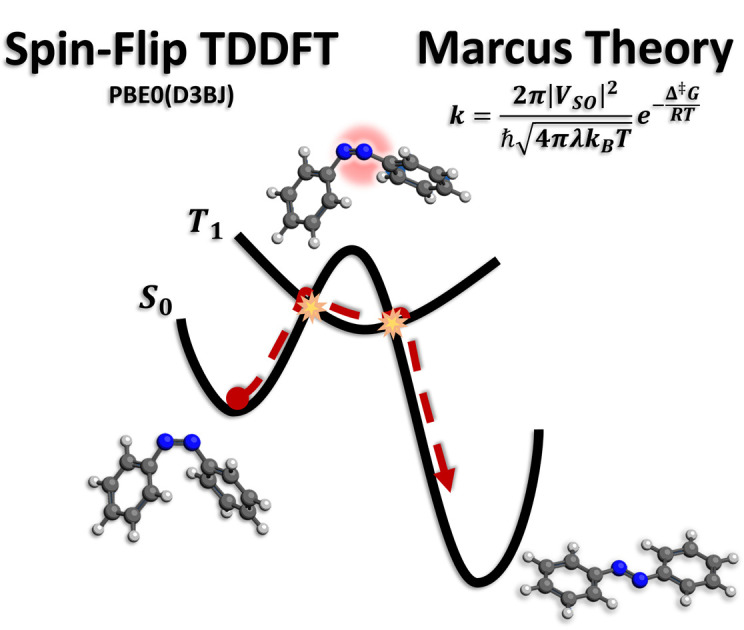

## 1 Introduction

**Fig. 1:**
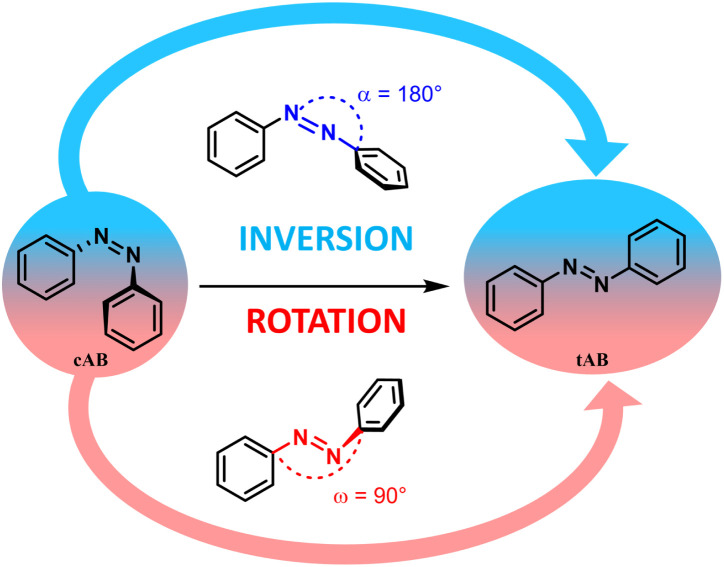
Reaction pathways for the back isomerization mechanism. α (in blue) is the NNC bending angle involved in the inversion, and ω (in red) is the dihedral angle involved in the rotation.

Modern society faces numerous energy-related challenges, especially in the transition from fossil fuels to renewable energy sources. To this end, many technologies have been developed, such as solar panels to produce electricity, and batteries to store it. To avoid inherent charge losses, an interesting new approach relies on the storage of energy in chemical structures. To do so, photo-switchable molecules that can isomerize by capturing a photon from the sun are used. A metastable isomer is then created, in which the solar energy is stored; this is referred to as a MOlecular Solar Thermal (MOST) system. This technology has been widely studied over the last decade.^1–3^ Among the various existing photochromic systems, azobenzene and derivatives appear very promising.^1,4–6^ Azobenzene can photo-isomerize from a *trans* (*E*) stable isomer to a *cis* (*Z*) metastable isomer, as shown in Fig. 1. These compounds offer many advantages, such as a low production cost, a high photo-isomerization quantum yield (44%), a large half-lifetime of the metastable form (about 4 days) and a decent storage energy density (0.20 MJ kg^−1^, or 36 kJ mol^−1^).^7^ Photo-isomerization, which can be considered the chemical analogue of the charging process in a battery, has been extensively studied.^1,4,8^ However, thermal back-isomerization, which can be considered the discharging process, has been largely overlooked. To the best of our knowledge, only a few studies have focused on this process.^9–14^ It is commonly accepted that the thermal back-isomerization can occur *via* two distinct mechanisms; the first involves the opening of the NNC angle, referred to as the inversion mechanism, while the second one proceeds *via* a rotation of a phenyl group around the double bond between the two nitrogen atoms and is referred to as the rotation mechanism (Fig. 1).

**Fig. 2:**
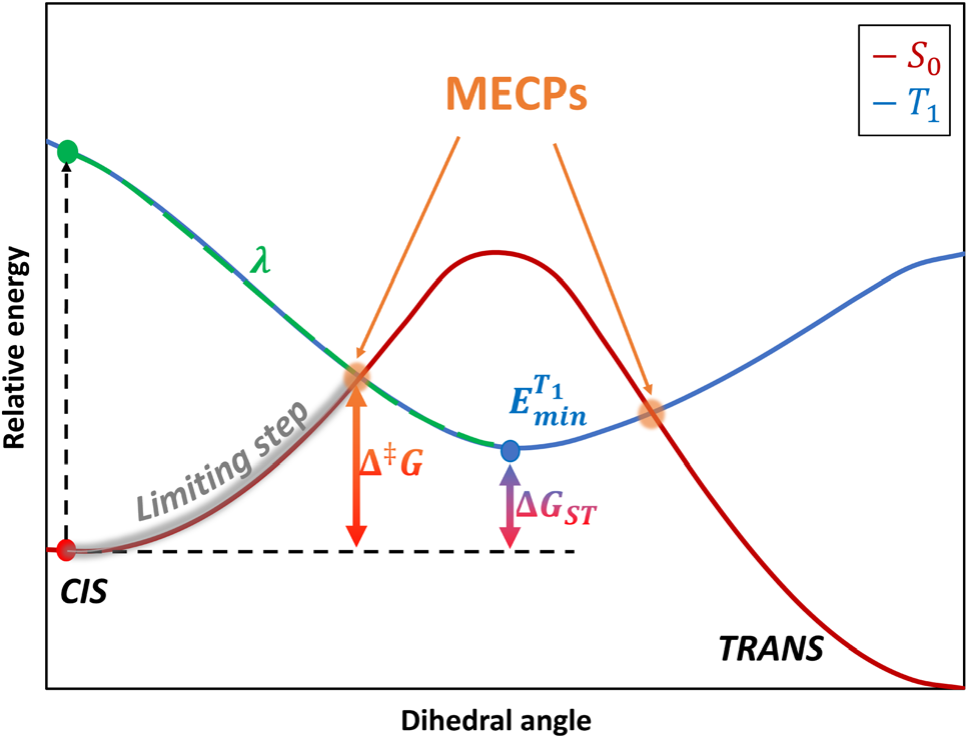
Schematic representation of the electronic states involved in the actual minimum energy reaction path. The S_0_ and T_1_ are represented as red and blue curves, respectively. MECP stands for the minimum-energy crossing-point. The limiting step considered in this study is highlighted in grey. Parameters entering into the WKB and Marcus rate equations are displayed on the scheme, *i.e. λ*, Δ^‡^*G*, and Δ*G*_ST_.

The minimum energy reaction path (MERP) was typically searched by DFT calculations, to describe the inversion mechanism.^15^ However, an inconsistency was pointed out recently: the activation entropy, Δ^‡^*S*, is calculated to be positive (about +10 J mol^−1^ K^−1^) at the DFT level against a negative experimental value for azobenzene (about −50.2 J mol^−1^ K^−1^).^16,17^ The experimental rate constant was well reproduced from a standard Eyring theory based on molecular parameters calculated at the DFT level, whereas Hartree-Fock and post-HF methods (such as MP2) were not able to provide the right order of magnitude.^17^ This discrepancy in the sign and amplitude of Δ^‡^*S*, known as the “entropy puzzle”, was studied recently in detail by Reimann and coworkers.^10^ Their highly correlated calculations suggest that the actual mechanism for the thermal back-isomerization of azobenzene is not an inversion but is rather based on a multi-state rotational process. Instead of a classical chemical reaction occurring on a single potential energy surface (PES), it was found that the lowest triplet state, T_1_, intersects with the ground state, S_0_ (Fig. 2). Estimating the rate constant thus requires accounting for a possible transition between these two states if the intersystem crossing is sufficiently fast (in other words, a non-adiabatic transition state description). The importance of the T_1_ state was supported experimentally by observing, in a few instances, an external heavy-atom effect on the kinetics of back-isomerization.^10,13^ Since the mixing between singlet and triplet states involves a spin–orbit coupling, adding heavy elements is expected to enhance this coupling, which is proportional to the fourth power of the nuclear charge.^18^

The electronic nature of this triplet state can be understood from a simple molecular orbital representation. During the rotation, the azo-π bond is broken since the 2p_*z*_ orbitals of each nitrogen no longer overlap, which can promote a biradical form either in a singlet or triplet state. According to Hund's rule, the lowest triplet state will be lower in energy than the lowest singlet state at 90°, as presented in Fig. 2. A drawback of DFT is that it fails to locate a transition state for the rotation mechanism by systematically converging to the transition state associated with the inversion mechanism.^10,13^ A possibility to overcome this problem is to perform a constrained optimization by freezing the NNC angles.^14^ This discrepancy was explained by the fact that the ground state and the first singlet excited state S_1_ mix near 90°: this mixing can be quantitatively described only with multi-reference methods, or at least with a method that can capture the multi-configurational physics of the problem.^10^ A quasi-exact value for the activation entropy can thus be recovered by combining highly correlated methods with a formalism taking into account the amplitude of the spin orbit coupling at the crossing of the S_0_ and T_1_ states, such as the semi-classical Wentzel–Kramers–Brillouin (WBK) theory.^10^

In order to efficiently explore the vast chemical space of potential MOST candidates, there is a need to reduce the computational effort while maintaining a good level of accuracy. In the search for an alternative approach for the coupling of highly correlated methods to the WBK theory used in ref. 10, the present work aims to investigate the relevance of coupling the results of spin-flip(SF) TD-DFT calculations with the WKB theory. SF-TDDFT is currently emerging as a fast and accurate method to be used in the presence of a conical intersection or to deal with a ground state whose wave-function cannot be simply represented with a single determinant.^19^ A secondary aim is to assess whether the use of the semi-classical Marcus theory to describe the limiting kinetic step can provide rates similar to those obtained with WBK. It must be noted that Marcus theory has already been used to compute the rate of intersystem crossing processes by relying on three easily accessible molecular parameters (*i.e.*, the reorganization energy, the spin–orbit coupling, and the free energy of the reaction). This approach will be first applied to the prototypical azobenzene molecule and will then be extended to other systems.

## 2 Theoretical background

We have first performed a benchmark of different DFT functionals to reproduce the potential energy curves of the S_0_ and T_1_ states taking CASPT2 results as the reference. We also employed the D3BJ dispersion correction, since DFT functionals usually do not correctly reproduce the inter/intramolecular van der Waals interactions.^20^ All the calculations were performed using Q-Chem 6.3 at the SF-TDDFT and DFT level and OpenMolcas 25.06 for the CASSCF/CASPT2 calculations. The spin-flip approach relies on the linear-response time-dependent DFT (LR-TDDFT) theory, which is well described by Casida's equation:1
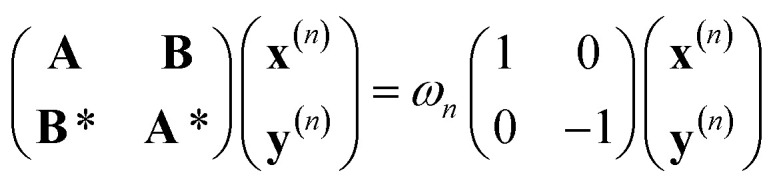
where *ω* is the excitation energy and **x** and **y** are vectors containing the excitation and de-excitation coefficients for each excited state. In the classical formalism of TDDFT, the reference determinant corresponds to the ground-state solution of the Kohn–Sham equation. The spin-flip approach takes as the reference state a well-behaved high-spin state, typically the T_1_ state (*m*_S_ = 1), such that lower-spin states (*e.g.* the S_0_ state) are recovered by a simple excitation with a spin-flip process from this high-spin reference state. In Casida's equation (eqn (1)), the **A** and **B** matrices have the following expression within classical TDDFT (using Mulliken's notation):

2 *A*_*ia*_^*jb*^ = *δ*_*ij*_*δ*_*ab*_(*ε*_*a*_ − *ε*_*i*_) + (*ia*|*f*_H_|*jb*) + (*ia*|*f*_xc_|*jb*)

3 *B*_*ia*_^*jb*^ = (*ia*|*f*_H_|*bj*) + (*ia*|*f*_xc_|*bj*)

where *f*_H_ and *f*_xc_ are the Hartree and exchange-correlation kernels. In the presence of a spin-flip transition, the matrices **A** and **B** (eqn (2) and (3)) further simplify into the following form for a hybrid functional:*A*_*iā*_^*jb̄*^ = *δ*_*ij*_*δ*_*āb̄*_(*ε*_*ā*_ − *ε*_*i*_) − *C*_HF_(*ij*|*f*_H_|*āb̄*)*B*_*iā*_^*jb̄*^ = −*C*_HF_(*ib*|*f*_H_|*āj̄*)with *C*_HF_ as the fraction of exact exchange included in the hybrid functional. The upper bars indicate the *β* spin-orbitals, as in ref. 19. Thus, from a single-reference formalism, we can generate a multi-configurational wavefunction that can be used to describe situations where static correlation is important, *e.g.* bond breaking and conical intersection.^19^ The main problem of SF-TDDFT is that it may suffer from spin-contamination; in such a case, spin-adapted (SA) SF-TDDFT or mixed-reference (MR) SF-TDDFT can be used to significantly reduce spin-contamination. In all our calculations, the maximum spin-contamination was 0.1; this value is reasonably small, and we thus consider that it did not interfere significantly with the results.

As mentioned above, it is necessary to go beyond the Eyring transition state theory (TST) and turn to a Non-Adiabatic TST (NA-TST); this formalism has already been applied to spin-forbidden reactions.^21^ If we consider two crossing potential energy surfaces associated with S_0_ and T_1_, as shown in Fig. 2, the hopping probability from S_0_ to T_1_ at the crossing point can be calculated from the Landau–Zener (LZ) theory as:^21–24^

4 *p* = (1 − *P*_LZ_)(1 + *P*_LZ_)

In eqn (4), *p* considers that the hopping from one diabatic state to another can take place along the first pass at the crossing point or at the second pass when going back to the equilibrium geometry of the initial state, and5
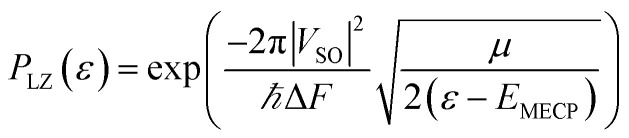
*P*_LZ_ is the Landau–Zener probability of hopping from one adiabatic state to another during the first pass. In eqn (5), *V*_SO_ is the spin–orbit coupling, necessary to promote the interaction between the singlet and triplet states, Δ*F* = |*F*_S_0__ − *F*_T_1__| is the relative slope of the two surfaces at the crossing point, *µ* is the reduced mass of the vibration orthogonal to the crossing seam along the hopping coordinate, *E*_MECP_ is the energy of the minimum-energy crossing-point (MECP), and the energy *ε* lies in the energy gap between the two adiabatic surfaces. From this equation, the semi-classical Marcus equation (eqn (6)) can be derived by relying on the parabolic shape of the potential energy surfaces of reactants and products:^25^6
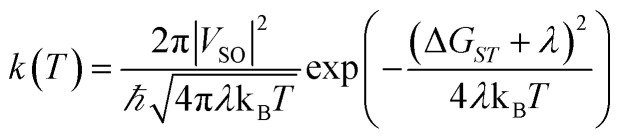
where *λ* is the reorganization energy accounting for the change in geometry going from the equilibrium geometry in the ground state to that in the triplet state (T_1_). Δ*G*_ST_ is the free energy difference between the S_0_ and T_1_ minima. In the LZ theory, the hopping probability is equal to zero for energies below the energy of the MECP. An alternative formalism allowing for tunneling effects is to consider the following equation:^21,23^7
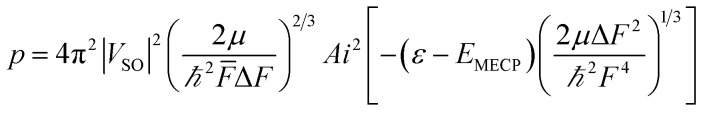
where *Ai* denotes the Airy function and *F̄* is the average of the slopes. We can approximate this equation to:^23^8
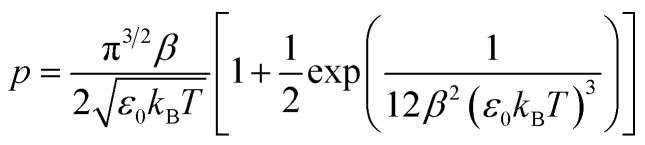

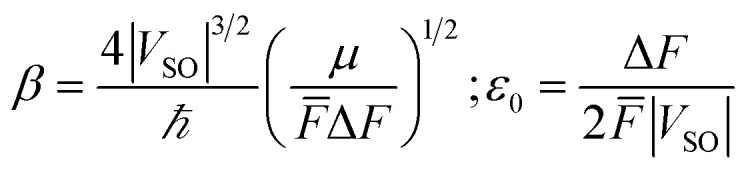
which is also known as the Wentzel–Kramers–Brillouin (WKB) approximation.^10,23^ All the parameters needed (*µ*,Δ*F*,*F̄*) are calculated using the GLOWFreq suite.^26^ Assuming the S_0_ → T_1_ crossing as the rate-determining step, we can use *p* from eqn (8) as the transmission coefficient, *γ*(*T*) = *p*, in the Eyring equation:9
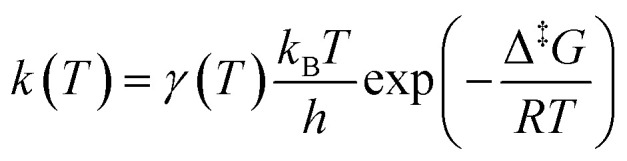
Using the definition Δ^‡^*G* = Δ^‡^*H* − *T*Δ^‡^*S*, eqn (9) can be transformed to obtain an equation linear in 1/*T*:10
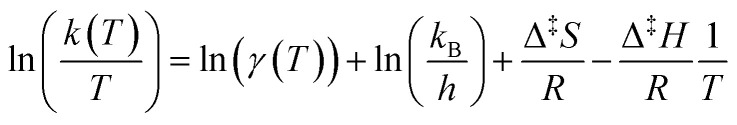


By plotting the left-hand side of eqn (10) with respect to 1/*T*, and neglecting the temperature dependence of *γ*(*T*), one obtains a straight line with slope −Δ^‡^*H*/*R* and intercept 
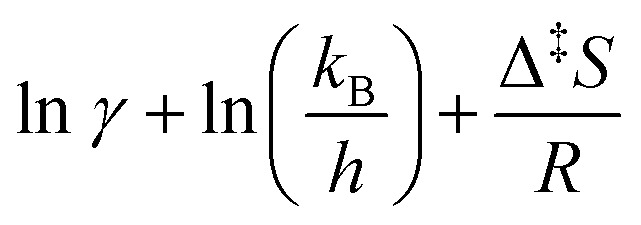
. This is generally known as an Eyring plot, and it is often used in the analysis of experimental data, with the further assumption of *γ* = 1.

However, as pointed out by Reimann *et al.*^10^ this latest assumption is no longer valid if spin-forbidden reactions are involved, for which *γ* ≪ 1. In this case, the apparent “measured” entropy Δ^‡^*S̄* = Δ^‡^*S* + *R* ln *γ* can acquire large negative values, even though the real entropy is probably small, as predicted by QM calculations.^14,17^ Concerning the non-adiabatic rotation mechanism, we computed Δ^‡^*G*, which corresponds to the free energy difference between the *cis* isomer and the MECP, and obtained Δ^‡^*H* and Δ^‡^*S* from an Eyring plot (eqn (10)). For the adiabatic rotation and inversion mechanisms, in the absence of intersystem crossing between the S_0_ and T_1_ states, Δ^‡^*H* and Δ^‡^*S* were directly obtained from frequency calculations at the *cis* and transition state geometries.

The spin–orbit coupling (SOC), *V*_SO_ in eqn (6) and (8), is a relativistic interaction between the spin angular momentum and the orbital angular momentum of an electron. This interaction is described by the Breit–Pauli spin–orbit operator, as implemented in Q-Chem 6.3:^18,27^11
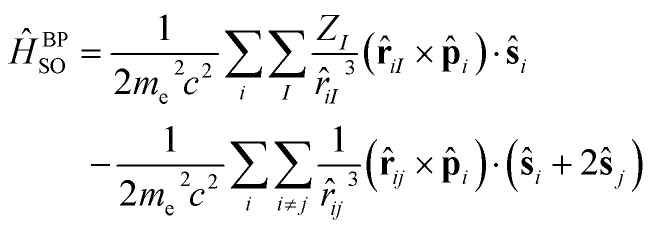


where *i* and *j* are the labels for electrons, *Z*_*I*_ is the charge of the nucleus *I*, *m*_e_ is the electron mass, and *c* is the speed of light. The first term (eqn (11)) corresponds to the one-electron part, which is the interaction of the spin of the electron with its orbital angular momentum. The second term (eqn (11)) is the two-electron contribution which accounts for the electron–electron interaction. In Q-Chem 6.3, the two-electron part is treated *via* a mean-field approach.^27,28^

## 3 Results and discussion

**Fig. 3:**
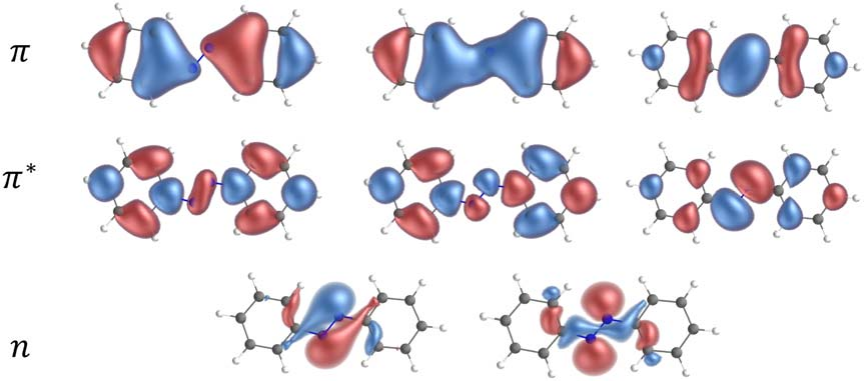
Orbitals involved in the active space for the CASPT2/ANO-RCC-VDZP calculations on *trans* azobenzene.

The first step in the present work was to carefully validate a functional to be used with SF-TDDFT. It has been reported that BHHLYP is a good starting point within the spin-flip method, due to its high exchange contribution, which is important for this kind of calculation involving singlet and triplet excited states.^29^ Nevertheless, we decided to perform a comparison of different functionals in order to find the one that best reproduces the results of the complete active space perturbation theory (CASPT2) method, *i.e.*, a highly-correlated multi-determinant approach taken here as the reference. We used the extended-multi state CASPT2 (XMS-CASPT2) formalism and the double zeta ANO-RCC-VDZP basis set with an active space (AS) of 10 electrons in 8 orbitals (Fig. 3).

The AS consists of two occupied lone-pair orbitals localized on the two nitrogen atoms and all the π orbitals except for the two most bonding and antibonding ones. The AS was selected following the procedure proposed by Aleotti *et al.*^14^ We performed a relaxed scan of the CNNC dihedral angle with steps of 10° for the ground state, and the triplet state energies were evaluated from single-point calculations at the ground state geometries. Our results are consistent with previously reported calculations.^9,14^ We repeated the same procedure for a set of 8 DFT functionals with the 6-311G(d,p) basis set, as shown in the SI (Fig. S1). Our approach, consisting of exploring different rungs of Jacob's ladder in DFT, (*i.e.*, GGA, hybrid, range-separated, *etc.*) showed that PBE0(D3BJ)/6-311G(d,p) calculations are those that best reproduce the ground and triplet state curves provided by the CASPT2 calculations.

**Fig. 4:**
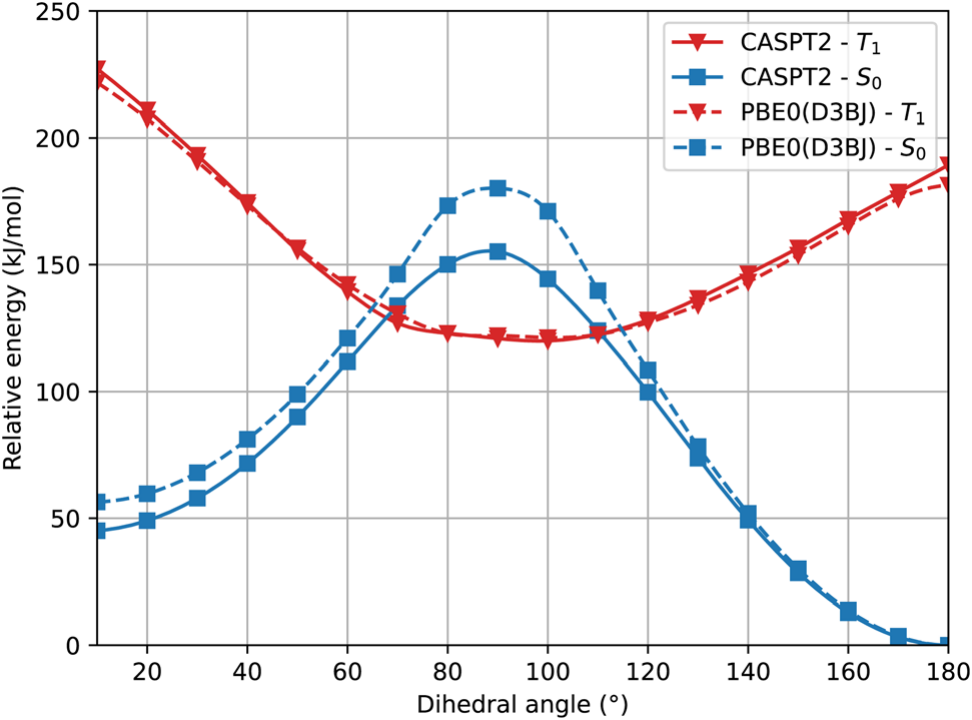
Relaxed scan of the S_0_ and T_1_ states in blue squares and in red triangles, respectively. The dotted lines represent the SF-TDDFT PBE0(D3BJ) calculations and the full lines represent the CASPT2 results.

**Fig. 5:**
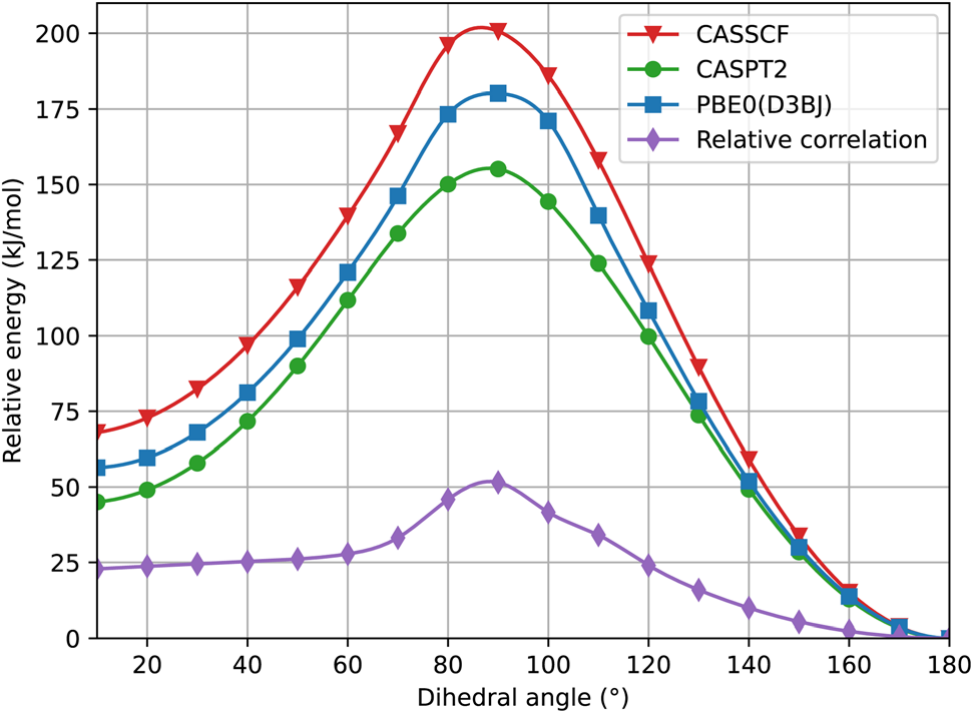
Torsional energy curve of the S_0_ state with CASSCF (red line, with triangles), CASPT2 (green line, with dots) and PBE0(D3BJ) at the SF-TDDFT level of theory (blue line, with squares). The purple line with diamonds corresponds to the correlation energy relative to the lowest value, which is at 180°. The CASSCF energy is calculated at the CASPT2 relaxed geometry.

In Fig. 4 we show a comparison of the relative singlet and triplet state energies as a function of the dihedral angle, for two methods, with respect to the singlet state reference energies arbitrarily set as those predicted at 180°. The agreement for the triplet state is excellent at any angle, while for S_0_ there is a general overestimation of the energy along the pathway in the S_0_ state at the PBE0 level, compared to the CASPT2 results. We attribute this overestimation in part to the lack of dynamic correlation in DFT methods. Not surprisingly given the choice of our reference energies, for dihedral angles close to 180°, we observe that the energy difference between CASPT2 and SF-TDDFT curves is very small, with a mean value of *c.a.* 1.5 kJ mol^−1^ for angles ranging from 130° to 180°. Instead, the discrepancy is larger around the *cis* form: for angles between 10° and 60°, the deviation is about 10 kJ mol^−1^ while for angles between 70–110°, the deviation amounts to 18 kJ mol^−1^. This large deviation probably occurs because, at these angles, the two phenyl rings are very close to each other, a situation where electron correlation is expected to be more important. To further validate this hypothesis, we examined how the ground state energy curve is affected by the perturbation theory treatment by comparing CASSCF *versus* CASPT2 results; in CASPT2 calculations, the dynamic correlation is recovered by perturbation theory, exactly in the same way as in the Møller–Plesset approach at the second order (MP2 – perturbation theory applied to a HF single-reference wavefunction). Notably, the correlation energy accounts for only 0.33% of the total energy, which is consistent with the validity of a perturbative treatment, where the perturbation is expected to remain small. Fig. 5 clearly shows that the PBE0(D3BJ) potential energy curve lies between the CASSCF and CASPT2 curves, indicating that the recovery of dynamic electron correlation plays a significant role. In purple, we report the correlation energy with respect to the *trans* isomer, for which the correlation energy is minimal: the curve peaks at 90°, showing that dynamic correlation is most significant at this dihedral angle, reaching a value of 50 kJ mol^−1^, which is not negligible.

**Fig. 6:**
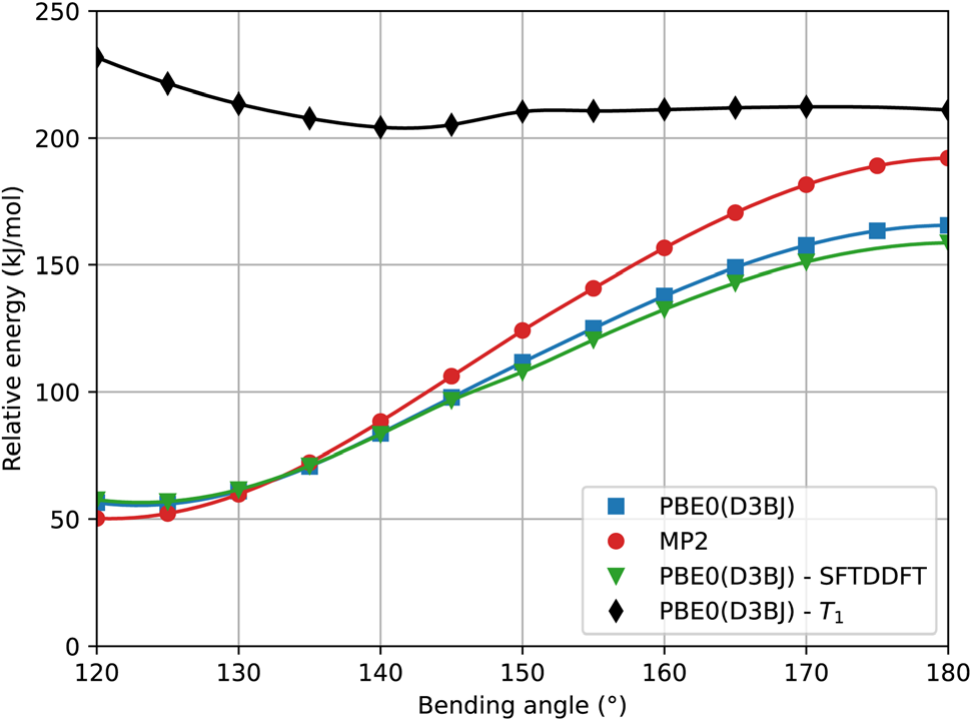
Pathway of the *Z* → *E* inversion mechanism following the bending angle NNC at different levels of theory. The basis set used for the MP2 calculations is cc-pVTZ.

We have discussed so far the rotation mechanism, which is commonly assumed to be the minimum-energy reaction path. For completeness, we also analyzed the energy profile associated with the inversion mechanism. From Fig. 6, it is clear that standard DFT and the spin-flip approach give very similar results, which is in agreement with the hypothesis that the inversion path can be described with a single-reference method. Using the Møller–Plesset second-order perturbation theory (MP2) as the reference method, our DFT approach underestimates the activation barrier mostly near the transition state. However, CASPT2 results appear to be in better agreement with DFT (see Table 1).

**Table 1:** Activation parameters for the *Z* → *E* isomerization calculated with the WKB or Marcus expression for the non-adiabatic rotation and with the Eyring equation for classical rotation and inversion. Enthalpies are in kJ mol^−1^ and entropies in J mol^−1^ K^−1^. Underlined values are taken from ref. 14 and t.b.e (theoretical best estimate), was calculated in ref. 10 as the arithmetic average of CCSDT, CCSDT(Q) and MRCI + Q energies. The values of *k* and *γ* were calculated at 298 K using the SF-PBE0(D3BJ) approach for each mechanism. The value of *λ*, used in the Marcus equation, was calculated to be *λ* = 119 kJ mol^−1^

	Non-adiabatic rotation						Rotation		Inversion	
	WKB (eqn (8))		Marcus (eqn (6))		Adapted Marcus (eqn (13))		Eyring (eqn (9))		Eyring (eqn (9))	
	Δ^‡^*H*	Δ^‡^*S̄*	Δ^‡^*H*	Δ^‡^*S̄*	Δ^‡^*H*	Δ^‡^*S̄*	Δ^‡^*H*	Δ^‡^*S*	Δ^‡^*H*	Δ^‡^*S*
CASPT2	78.9	−39	70	−31	82.0	−53	100.5	−13.5	126.7	2.1
PBE0(D3BJ)	90.2	−32	59.3	−45	88.3	−53	124.2	−27	119.2	4.9
MP2	—	—	—	—	—	—	—	—	137.1	16.9
*k* (298 K)	4.9 × 10^−5^ s^−1^		9.8 × 10^−1^ s^−1^		4.4 × 10^−6^ s^−1^		4.0 × 10^−11^ s^−1^		1.4 × 10^−8^ s^−1^	
γ (298 K)	4.98 × 10^−2^		1.5 × 10^−2^		8.35 × 10^−3^		1		1	
Experimental^16^	88.6	−50.2	*k* = 8.5 × 10^−6^ s^−1^							
t.b.e^10^	95 ± 4	−38 ± 2	—	—	—	—	118 ± 7	−8	124 ± 3	11

The next step is to determine the activation parameters, *i.e.* Δ^‡^*H* and Δ^‡^*S*, of the back-isomerization process. There are thus three possible paths: the inversion mechanism, the purely ground state rotation mechanism, and the rotation mechanism combined with a spin-forbidden transition into the T_1_ state. The latter was referred to as non-adiabatic rotation in a recent article.^30^ Regarding the non-adiabatic rotation, using the WKB approximation (eqn (8)) fed by the results of highly correlated CCSD calculations, Reimann *et al.* found theoretical values in really good quantitative agreement with the experiment;^10^ however, CCSD is still a single-reference method, which implies that it will suffer from the same cusp problem as normal DFT (lack of strong correlation) when the dihedral angle is around 90°. We also employed eqn (8) and found a nice match between the values in ref. 10 and ours (see Table 1) when injecting parameters obtained at the spin-flip PBE0(D3BJ) level, and in particular a negative value for the entropy of activation (−32 J mol^−1^ K^−1^), strengthening the idea that its sign can be associated with a spin-forbidden process.^21^ The larger the spin–orbit coupling, the more pronounced these spin-forbidden effects become; azobenzene exhibits a spin–orbit coupling of approximately 15 cm^−1^, which is relatively large compared to typical organic conjugated systems.^31^ This coupling was calculated at the MECP geometry. Regarding the adiabatic rotation and inversion pathways, we evaluated the activation enthalpy and entropy based on frequency calculations by determining the partition function^32^ in the ground and transition state geometry. From the comparison of WKB enthalpies, entropies and rates with experimental ones (Table 1), it is clear that this approach performs well in describing the non-adiabatic rotational mechanism. Indeed, it yields the correct order of magnitude for the rate constant (order of 10^−5^ s^−1^) and provides activation parameters that are consistent with experimental data as well as with other theoretical methods. However, when the Marcus equation, as formulated in eqn (6), is applied (Table 1), although the activation entropy is well reproduced, it shows a significant discrepancy in the activation enthalpy (59.3 *vs.* 90.2 kJ mol^−1^). This inaccuracy originates from the Δ*G*_ST_ term: a more detailed analysis of the two key parameters in the Marcus equation – namely the reorganization energy *λ* and Δ*G*_ST_, the free-energy difference between the triplet and singlet minima—reveals that the reorganization energy is in good agreement with the CASPT2 calculations. In contrast, the energy of the *cis* isomer is overestimated at the DFT level, leading to an incorrect estimation of Δ*G*_ST_.

In the Marcus equation, Δ*G*_ST_ and *λ* are employed to estimate the transition state energy without actually measuring or calculating it directly with quantum chemistry methods:12
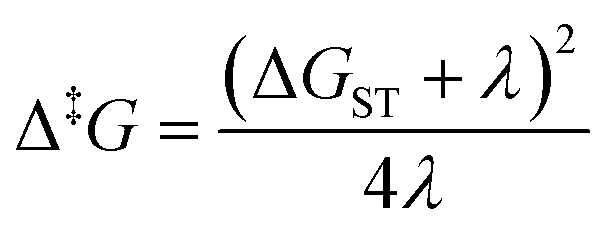


This formula further assumes that both potential energy surfaces are perfect parabolas with the same curvature, which is not necessarily the case. We have here adopted an alternative formulation of eqn (6):13
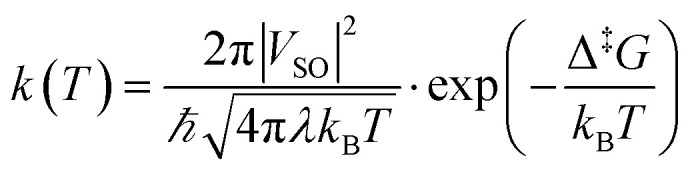


in which, while retaining the Marcus pre-exponential factor, featuring the spin–orbit coupling, *V*_SO_, we replaced the exponential term in eqn (6) with that used in the Eyring equation (eqn (9)). Δ^‡^*G* is estimated from energies almost equally overestimated at the DFT level. This modified expression is employed in Table 1 to compute the kinetic and activation parameters. It performs remarkably well, yielding activation parameters that are very close to the experimental values (88.3 *vs.* 88.6 kJ mol^−1^ for the enthalpy and −53 *vs.* −50.2 J mol^−1^ K^−1^ for the entropy) and to those predicted by WKB theory (90.2 kJ mol^−1^ and −32 J mol^−1^ K^−1^) at a much higher computational cost. Eqn (13) therefore offers a faster, more efficient, and simpler route to determine the rate constant (and the associated activation parameters) for the non-adiabatic rotational mechanism.

**Fig. 7:**
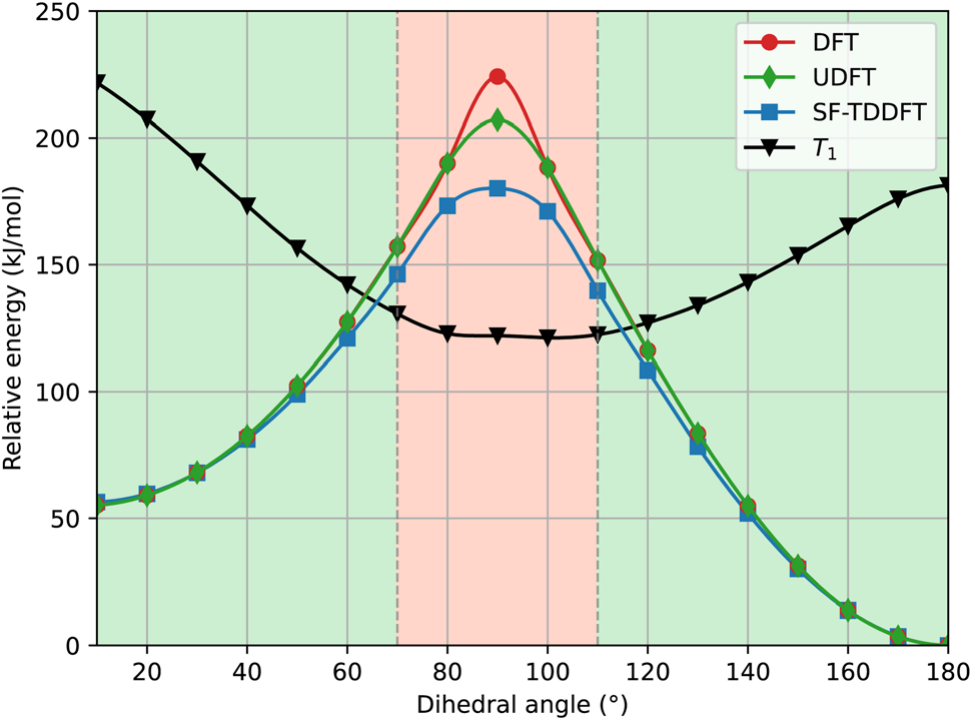
Single-point energy calculations at the DFT and UDFT level of theory from SF-TDDFT-computed relaxed geometries. In the green area, there is a perfect match between the three methods. In the red area, there is a significant deviation. The T_1_ state is represented by the black curve, computed at the DFT level of theory. All calculations were performed at the PBE0(D3)/6-311G(d,p) level of theory.

We have also assessed the influence of the S_0_/S_1_ mixing in the calculations. To do so, we performed single-point energy calculations with DFT and unrestricted (Broken-Symmetry, BS) DFT at geometries obtained from a SF-TDDFT scan of the singlet ground state. As depicted in Fig. 7, there is a perfect match of all three methods close to the *cis* and *trans* minima (green area) and a significant deviation (about 44 kJ mol^−1^ for DFT and 27 kJ mol^−1^ for UDFT) only in the TS region (red area). This demonstrates the importance of the SF-TDDFT to take into account the multi-configurational nature of the transition state, which in this case is important due to the strong mixing between the S_0_ and S_1_ states. Regardless, it is worth stressing that both MECPs between the S_0_ and T_1_ states are located in the green area of Fig. 7, which indicates that one could use single reference methods, such as classical DFT, to calculate the parameters needed for evaluating the rate constant *via* the WKB or Marcus equations. With the latter approach, we can derive (see the SI) an expression for the transmission coefficient used in the Eyring equation,14
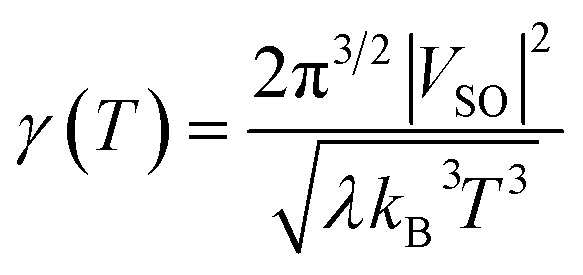
which is definitely simpler to evaluate than that in the WKB formalism (eqn (8)), since we only need the reorganization energy *λ* and the spin–orbit coupling *V*_SO_. We also tried the Marcus–Levich–Jortner approach to take into account tunneling effects, but this method relies on empirical parameters (*i.e.*, a single effective vibrational mode) which have a strong impact on the kinetic constant so that it is not recommended for quantitative estimates (see the SI for more details).

**Fig. 8:**
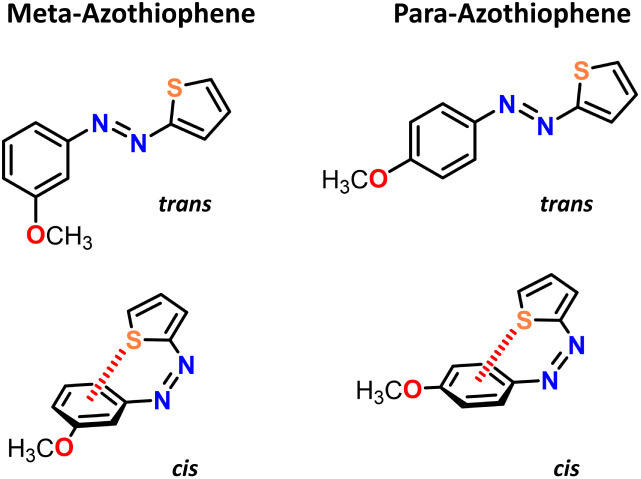
Chemical structures of *meta*-methoxy-azothiophene (mAth, left) and of *para*-methoxy-azothiophene (pAth, right).

Finally, as a proof of concept, we have applied the proposed methodology to two other azo derivatives: *para* and *meta* methoxy-azothiophene (pATh and mATh, respectively), as presented in Fig. 8. These two heterocyclic azo compounds are of particular interest because they include a thiophene moiety that can be involved in a weak intramolecular interaction between the sulfur atom and the phenyl group on the opposite side of the azo bond, thereby stabilizing the *cis* isomer. These systems were recently described by Martins *et al.*^30^ who found, based on CASPT2 calculations, that these two systems will back-isomerize *via* a mix of non-adiabatic and adiabatic rotations since the inversion pathway is too high in energy. We therefore tracked the inversion and rotation pathways and reported the calculated activation parameters in Table 2; they are found to be in good agreement with the results in ref. 30. Indeed, based on Table 2, the spin-flip PBE0(D3BJ) reproduces well the CASPT2 results for the non-adiabatic and adiabatic rotations. However, there is a non-negligible difference for the inversion mechanism (about 10 kJ mol^−1^), which can compete as well in our case; note that our calculated activation entropies for the inversion mechanism are closer to the experimental ones. Regarding the inversion terms, we observed that this mechanism preferentially occurs on the side of the azo double bond bearing the thiophene substituent (see the SI); however we feel more cautious about the individuation of the actually prevailing mechanism, since all enthalpies terms are close, but still the non-adiabatic rotation appears to be the minimum-energy path. It should also be kept in mind that if the mechanisms exhibit similar Δ^‡^*G* values, the adiabatic pathways will always be the fastest. One should also consider that the experimental values were obtained in acetonitrile, which is a polar solvent that might thus strongly influence the kinetics of the back-isomerization process. A more expensive approach (*e.g.* QMMM) could provide further information on how solvents affect the mechanisms. We also suggest an experimental investigation of the heavy-atom effect to obtain information on whether a triplet state is involved or not in the actual mechanism.

**Table 2:** Activation parameters calculated with WKB and the adapted Marcus equations for the non-adiabatic rotation and with the Eyring equation for classical rotation and inversion. SF stands for “Spin-Flip”, meaning SF-TDDFT evaluated at PBE0(D3BJ)/6-311G(d,p). Enthalpies are in kJ mol^−1^, entropies in J mol^−1^ K^−1^ and rate constants in s^−1^. We calculated the value of *λ* for each structure: *λ*_pATh_ = 135 kJ mol^−1^ and *λ*_mATh_ = 138 kJ mol^−1^. The value of the spin–orbit coupling is the following: *V*_SO_(pATh) = 13.5 cm^−1^ and *V*_SO_(mATh) = 12.9 cm^−1^. The values of *k* and *γ* were calculated at 298 K using the SF-PBE0(D3BJ) approach for each mechanism

	Non-adiabatic rotation				Rotation		Inversion	
	WKB (eqn (8))		Marcus (eqn (13))		Eyring (eqn (9))		Eyring (eqn (9))	
	Δ^‡^*H*	Δ^‡^*S̄*	Δ^‡^*H*	Δ^‡^*S̄*	Δ^‡^*H*	Δ^‡^*S*	Δ^‡^*H*	Δ^‡^*S*
CASPT2 (pATh)^30^	92.1	−39.7	—	—	105.3	−0.6	135.1	−0.2
CASPT2 (mATh)^30^	97.2	−43.2	—	—	111.8	0.2	138.1	−0.1
SF (pATh)	90.8	−32.0	88.4	−56.2	115.5	−4.9	100.0	−7.5
SF (mATh)	95.0	−31.0	92.4	−55.5	123.5	17.32	102.2	−2.9
*k*(298 K, pATh)	1.4 × 10^−5^		2.38 × 10^−6^		1.9 × 10^−8^		7.3 × 10^−6^	
*k*(298 K, mATh)	2.9 × 10^−6^		6.35 × 10^−7^		1.1 × 10^−8^		5.2 × 10^−6^	
*γ*(298 K, pATh)	3.5 × 10^−2^		5.8 × 10^−3^		1		1	
*γ*(298 K, mATh)	3.8 × 10^−2^		6.3 × 10^−3^		1		1	
Exp.^33^ (pATh)	86.6	−12.5	*k* = 3.9 × 10^−6^					
Exp.^33^ (mATh)	96.2	−12.5	*k* = 3.2 × 10^−7^					

## 4 Conclusions

The thermal back-isomerization of *cis* azobenzene is more complex than it first appears, since, although the inversion mechanism is well described using conventional DFT, the rotation mechanism exhibits pronounced multiconfigurational character at around 90°, where the S_0_ and S_1_ states strongly mix. Moreover, the emergence of a biradical character might lead to the involvement of a triplet state along the process. We found that the PBE0 functional with the D3BJ empirical dispersion and the 6-311G(d,p) basis set can reproduce highly correlated CASPT2 results by employing the SF-TDDFT formalism. This technique appears to be a powerful tool, as it reproduces highly correlated results in a shorter time. Subsequently, we showed that the Marcus equation can be used by considering the exponential term as described in the Eyring equation, which accounts for the activation energy (in our case, the energy required to reach the MECP), and retaining the actual pre-exponential factor of the Marcus equation. We obtain very accurate activation parameters (88.3 kJ mol^−1^ and −53 J mol^−1^ K^−1^), in excellent agreement with experimental data (88.6 kJ mol^−1^ and −50.2 J mol^−1^ K^−1^), which represents a major advance for the description of reaction mechanisms in azo compounds. Finally, we reproduced activation parameters for azothiophene derivatives as a proof of concept. This work is the first step towards exploring larger systems than azobenzene for the future, in order to understand how the molecular structure impacts the back isomerization kinetics.

## Author contributions

Ari Serez: writing – original draft, validation, methodology, investigation, conceptualization. Flavia Aleotti: methodology, writing – review & editing. Pascal Gerbaux: writing – review & editing, supervision, project administration, funding acquisition. Luca Muccioli: writing – review & editing, methodology. Jérôme Cornil: conceptualization, writing – review & editing, methodology, supervision, project administration, funding acquisition.

## Conflicts of interest

There are no conflicts to declare.

## Supplementary Material

SC-OLF-D6SC01578F-s001

## Data Availability

The data supporting this study are available from the corresponding author upon request. Supplementary information (SI): Benchmark of SF-TDDFT functionals, transmission coefficients derived from Marcus theory, details on Marcus–Levich–Jortner and mechanistic details on azothiophene derivatives. See DOI: https://doi.org/10.1039/d6sc01578f.
