# Supplementary material for: Mechanistic insights into azo compound back-isomerization from spin-flip time-dependent DFT combined with Marcus theory
Source: Chem Sci. 2026 Apr 27;17(24):12111–9. doi: 10.1039/d6sc01578f (PMC13170757; doi:10.1039/d6sc01578f)
Supplement: SC-017-D6SC01578F-s001 [file SC-017-D6SC01578F-s001.pdf]

Supplementary Information (SI) for Chemical Science.
This journal is © The Royal Society of Chemistry 2026

Supporting information for Mechanistic Insights into Azo Compound Back-Isomerization from Spin-Flip Time-Dependent DFT combined to Marcus Theory

Ari Serez,[†,‡] Flavia Aleotti,[¶] Pascal Gerbaux,[‡] Luca Muccioli,[¶] and Jérôme Cornil[*,†]

†*Laboratory for Chemistry of Novel Materials, University of Mons, Belgium*

‡*Organic Synthesis and Mass Spectrometry Laboratory, University of Mons, Belgium*

¶*Dipartimento di Chimica Industriale "Toso Montanari", University of Bologna, Via Gobetti 85, 40129 Bologna, Italy*

E-mail: Jerome.CORNIL@umons.ac.be

Azobenzene

Benchmarking of SF-TDDFT functionals

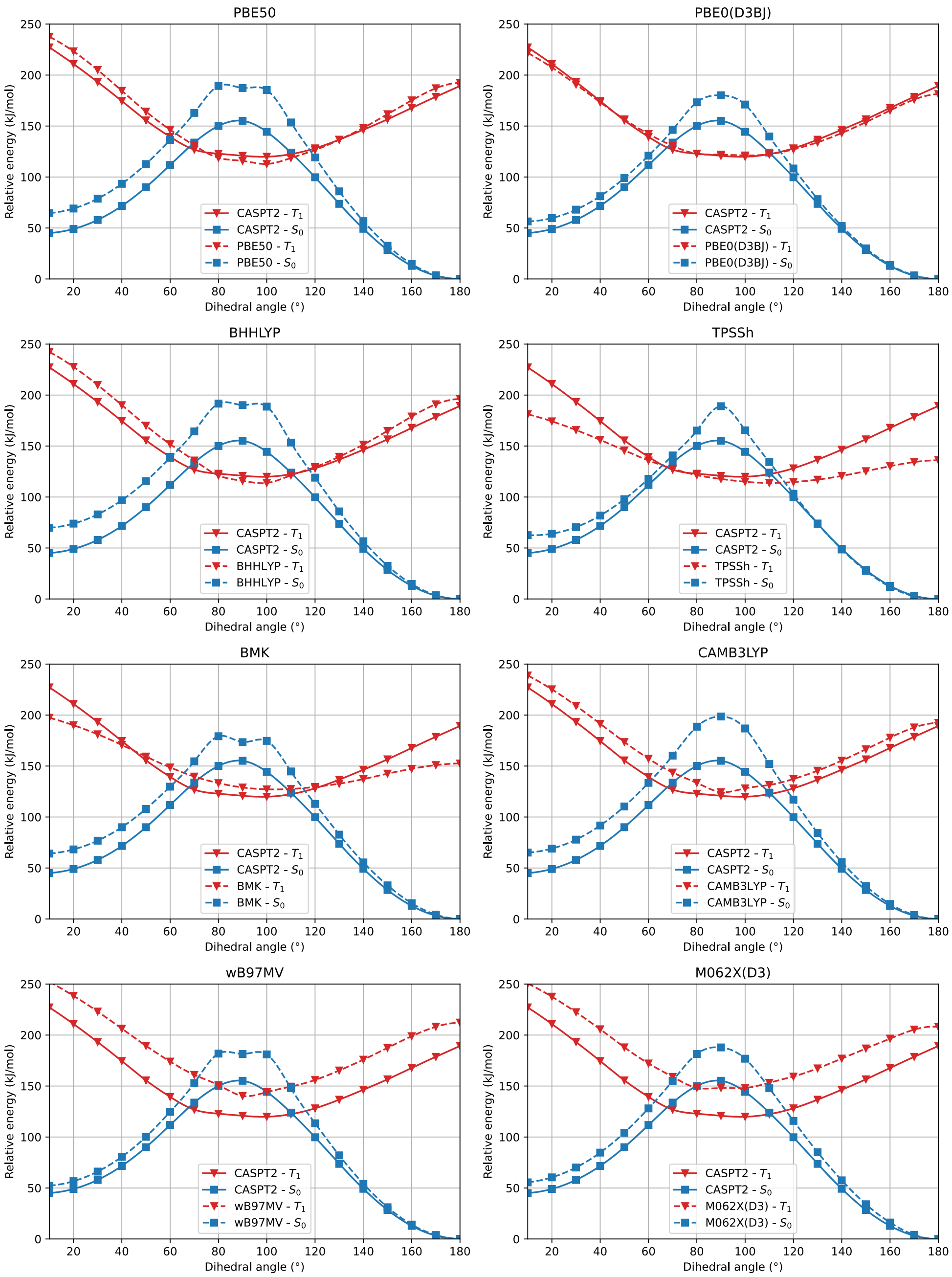

Figure S1: Benchmarking of 8 functionals against the CASPT2 reference. PBE0(D3BJ) appears to be the best functional in this group test, especially when comparing the T_1 curve.

Transmission coefficient derived from Marcus

$$\iff \quad \frac{k_B T}{h}\gamma e^{-\Delta G^{\ddagger}/RT} = \frac{2\pi |V_{SO}|^2}{\hbar\sqrt{4\pi\lambda k_B T}} e^{-(\Delta G_{ST}+\lambda)^2/4\lambda k_B T} \tag{1}$$

$$\iff \quad \frac{k_B T}{h}\gamma = \frac{2\pi |V_{SO}|^2}{\hbar\sqrt{4\pi\lambda k_B T}} \tag{2}$$

$$\iff \quad \gamma = \frac{h}{k_B T} \cdot \frac{2\pi |V_{SO}|^2}{\hbar\sqrt{4\pi\lambda k_B T}} \tag{3}$$

$$\iff \quad \gamma = \frac{2\pi^{3/2} |V_{SO}|^2}{\sqrt{\lambda k_B^3 T^3}} \tag{4}$$

(1) We equate the Eyring equation with the Marcus equation.

(1)→(2) Since the exponentials are equivalent (Eq. 11 in the main text), we can simplify the equation.

(3)→(4) By using the reduced Planck constant $\hbar = h/2\pi$, we can further simplify the equation.

Marcus-Levich-Jortner

The Marcus-Levich-Jortner (MLJ) theory is an improvement of Marcus equation incorporating tunneling effects. The one-effective mode version has the following form[1] :

$$k = \frac{\pi |V_{SO}|^2}{\hbar\sqrt{\pi\lambda_S k_B T}} \sum_{\nu=0}^{\infty} e^{-S} \frac{S^{\nu}}{\nu!} \exp\left(- \frac{(\Delta G^0 + \lambda_S + \nu\hbar\omega_{eff})^2}{4\lambda_S k_B T} \right) \tag{5}$$

In the equation above, λ_S corresponds to the reorganization energy of the solvent, S is the Huang-Rhys factor associated with the effective mode of frequency ω_{eff}. In the case of apolar media, λ_S ranges generally between 0.1 and 0.2 eV;[2] thus hereafter we consider that the molecular properties will not be strongly affected by solvation. In the following Table, we calculated k with various parameters.

Table 1: Values of k with different values of λ_S and ΔG^0. These values are given in eV and k is in s^{-1}.

λ_S / ΔG^0	0.5	0.6	0.7
0.1	$k \approx 7 \cdot 10^{-10}$	$k \approx 2 \cdot 10^{-15}$	$k \approx 1 \cdot 10^{-21}$
0.2	$k \approx 3 \cdot 10^{-5}$	$k \approx 2 \cdot 10^{-8}$	$k \approx 6 \cdot 10^{-12}$

From Table 1, values of k are ranging from 10^{-5} to 10^{-21}, depending on λ_S and ΔG^0, thus demonstrates a huge sensitivity to the input parameters. Based on these results, we prefer not using the MLJ equation for quantitative estimates of rate constants.

Azothiophene

We have first to find the global minimum of both *cis* and *trans* para/meta-methoxy - azothiophene (referred as pATh and mATh, respectively). These structures correspond to Fig 8 in the main text and the optimized structures are represented in Fig S2 below. Then, inversion and rotation pathways were investigated.

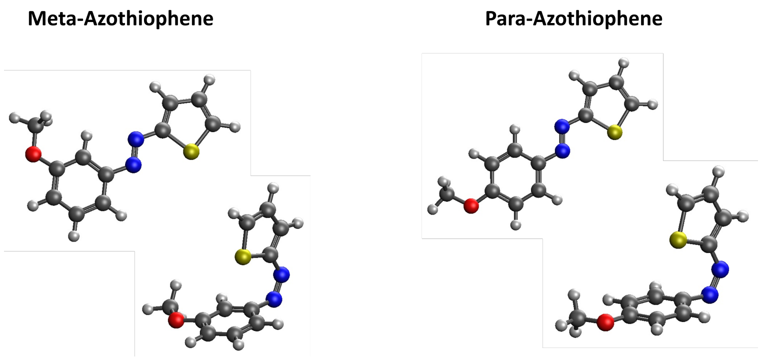

Figure S2: Chemical structures of meta-methoxy-azothiophene (left) and of para-methoxy-azothiophene (right). These structures correspond to the minimum-energy for each isomer, in their *trans* and *cis* configurations.

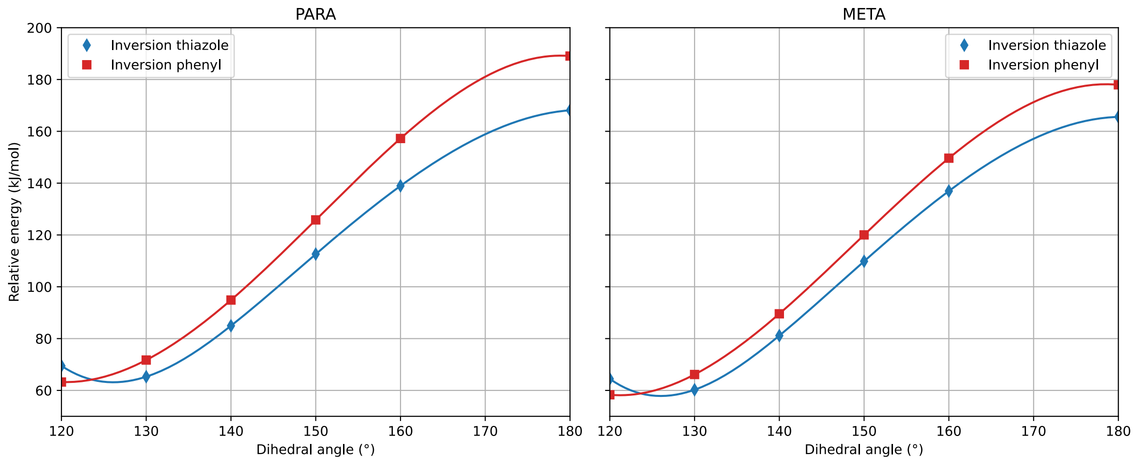

Figure S3: Inversion pathways for pATh on left and for mATh on the right.

From Fig S3, in both cases, the inversion related to the NNC angle involving the thiophene ring leads to a minimum-energy path. We thus only consider this path in our discussion.

The position of the methoxy substituent does not significantly influence the rotation pathway. The difference in activation enthalpy arises from the different stability of the *cis*

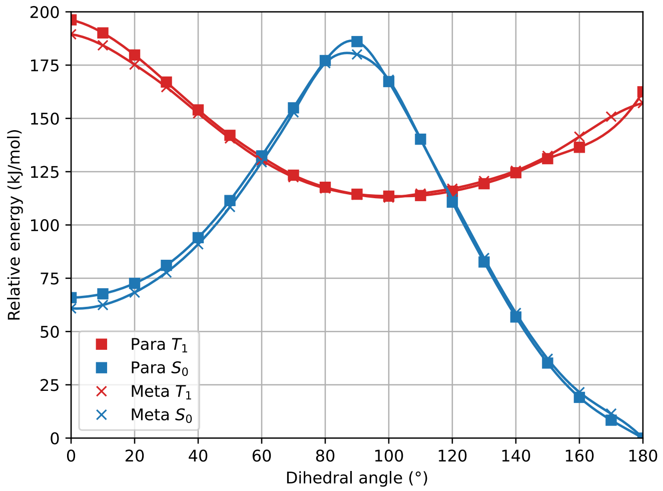

Figure S4: Rotation pathways for mATh and pATh.

form of the two compounds, depending on the position of the methoxy group. Since the mATh is less symmetric than its para counterpart, we have investigated both clockwise and anti-clockwise rotation around the N=N bond; a difference on the order of kT was observed and we selected the minimum-energy path.

References

(1) Subhajyoti, C.; Svante, H.; Dalvin D., M.-H.; Heidi P., H.; Kenneth A., J.; Junming, H.; Victor S., B. Electron Transfer Assisted by Vibronic Coupling from Multiple Modes. *Journal of Chemical Theory and Computation* **2017**, 6000–6009.

(2) Lemaur, V.; Steel, M.; Beljonne, D.; Brédas, J. L.; Cornil, J. Photoinduced charge generation and recombination dynamics in model donor/acceptor pairs for organic solar cell applications: A full quantum-chemical treatment. *Journal of the American Chemical Society* **2005**, *127*, 6077–6086.
